# The relative importance of macro versus micro geographical scale in explaining suicide variation in Seoul, South Korea 2014–2016

**DOI:** 10.1371/journal.pone.0273866

**Published:** 2022-09-09

**Authors:** Hwa-Young Lee, Rockli Kim, Soong-Nang Jang, Ichiro Kawachi

**Affiliations:** 1 Department of Global Health and Population, Harvard T.H. Chan School of Public Health, Boston, MA, United States of America; 2 Institute of Convergence Science, Convergence Science Academy, Yonsei University, Seoul, Korea; 3 Division of Health Policy and Management, College of Health Science, Korea University, Seoul, South Korea; 4 Department of Public Health Sciences, Interdisciplinary Program in Precision Public Health, Graduate School of Korea University, Seoul, South Korea; 5 Red Cross College of Nursing, Chung-Ang University, Seoul, South Korea; 6 Department of Social and Behavioral Sciences, Harvard. T.H. Chan School of Public Health, Boston MA, United States of America; University of New South Wales, AUSTRALIA

## Abstract

**Background:**

As ecological factors are getting attention as important determinants of suicide, it is important to identify the unit at which the largest variation exists for more tailed strategy to prevent suicide. We examined the relative importance of two administrative levels for geographic variation in the suicide rate between 2014–2016 in Seoul, the capital city of Korea.

**Methods:**

Two-level linear regression with Dongs (level 1) nested within Gus (level 2) was performed based on suicide death data aggregated at the Dong-level. We performed pooled analyses and then year-stratified analyses. Dong-level socioeconomic status and environmental characteristics were included as control variables.

**Results:**

The overall age- and sex- standardized suicide rate across all Dongs decreased over time from 24.9 deaths per 100,000 in 2014 to 23.7 deaths in 2016. When Dong and Gu units were simultaneously considered in a multilevel analysis, most of the variation in suicide rate was attributed to within-Gu, between-Dong differences with a contribution of Gu-level being small and decreasing over time in year (Variance partitioning coefficient of Gu = 5.3% in 2014, <0.1% in 2015 and 2016). The number of divorce cases per 100,000 explained a large fraction of variation in suicide rate at the Dong-level.

**Conclusions:**

Findings from this study suggest that ecological micro-area unit is more important in reducing the geographic variation in the suicide rate. More diverse ecological-level data needs to be collected for targeted area-based suicide prevention policies in Korea.

## Introduction

Every year, more than 800,000 die by suicide [1]. According to the most recent OECD data (2021), South Korea has the highest suicide rate among the OECD countries, with 24.6 suicides per 100,000 population, which is more than twice the OECD average (11.0 per 100,000) [2]. Suicide is prevalent across all age groups in Korea.

While a range of biological, psychological, social, and cultural factors have been proposed to explain population variations in suicide rates, much of the focus has been on individual-level factors such as history of major mental disorders (depression, bipolar disorder, and schizophrenia), trauma or adversity (divorce, separation, social isolation or childhood traumas) [3]. On the other hand, Emile Durkheim’s classic study (1897) approached suicide matter from an ecological perspective [4], arguing that spatial variations in suicide rates reflect the influence of underlying social forces in their area, such as norms and institutions. It has been reported that area-level characteristics such as population composition and availability of local resources can influence patterns of social interaction, and hence, lead to variations in suicide rate from area to area. Indeed, persistent and marked geographic variations in suicide rates have been observed between countries as well as within countries [5–7].

Reflecting this context, the third five-year national plan entitled “National action plan for suicide prevention” which was unveiled in 2018 in Korea underlined the importance of local government and community [8,9]. The plan specifically required each regional level (called “Si” or “Do”) and county/district-level (called “Gun” or “Gu”) government to submit a suicide prevention action plan tailored for their respective area [8,9]. To better inform these efforts, it is critical to understand the sources of geographical variation in suicide rates in Korea.

Seoul is the capital of South Korea and have two important administrative units, Gu and Dong. There are 25 autonomous Gu districts in Seoul, which are further divided into 242 administrative Dong sub-units. Each Gu is a basic local government entity that is responsible for assigned affairs and autonomous duties. Dong is more akin to neighborhood, with a more homogeneous composition of socioeconomic status and culture.

Only recently have a few studies in Korea begun to pay attention to area-level determinants of suicide rates. However, these studies only focused on examining the association between area characteristics and the suicide rate at a single selected level without considering the relative contribution of multiple alternative geographic levels. Relying only on a single geographic level often causes “missing unit problem”, meaning that the relative importance of any given geographic unit can only be accurately estimated when all levels that are considered to influence the outcomes are simultaneously included. Incomplete consideration of all relevant geographic scales may lead to over-or under- estimation of their contribution.

Policies in Korea are often performed at a specific unit due to administrative conveniences such as budget execution and mobilization of the workforce, which sometimes entails enforcing identically-designed interventions across the areas while ignoring the underlying heterogeneity. To maximize the policy effect in reducing health inequality, quantitative evidence identifying the unit at which the largest variation exists is much needed.

**Fig 1:**
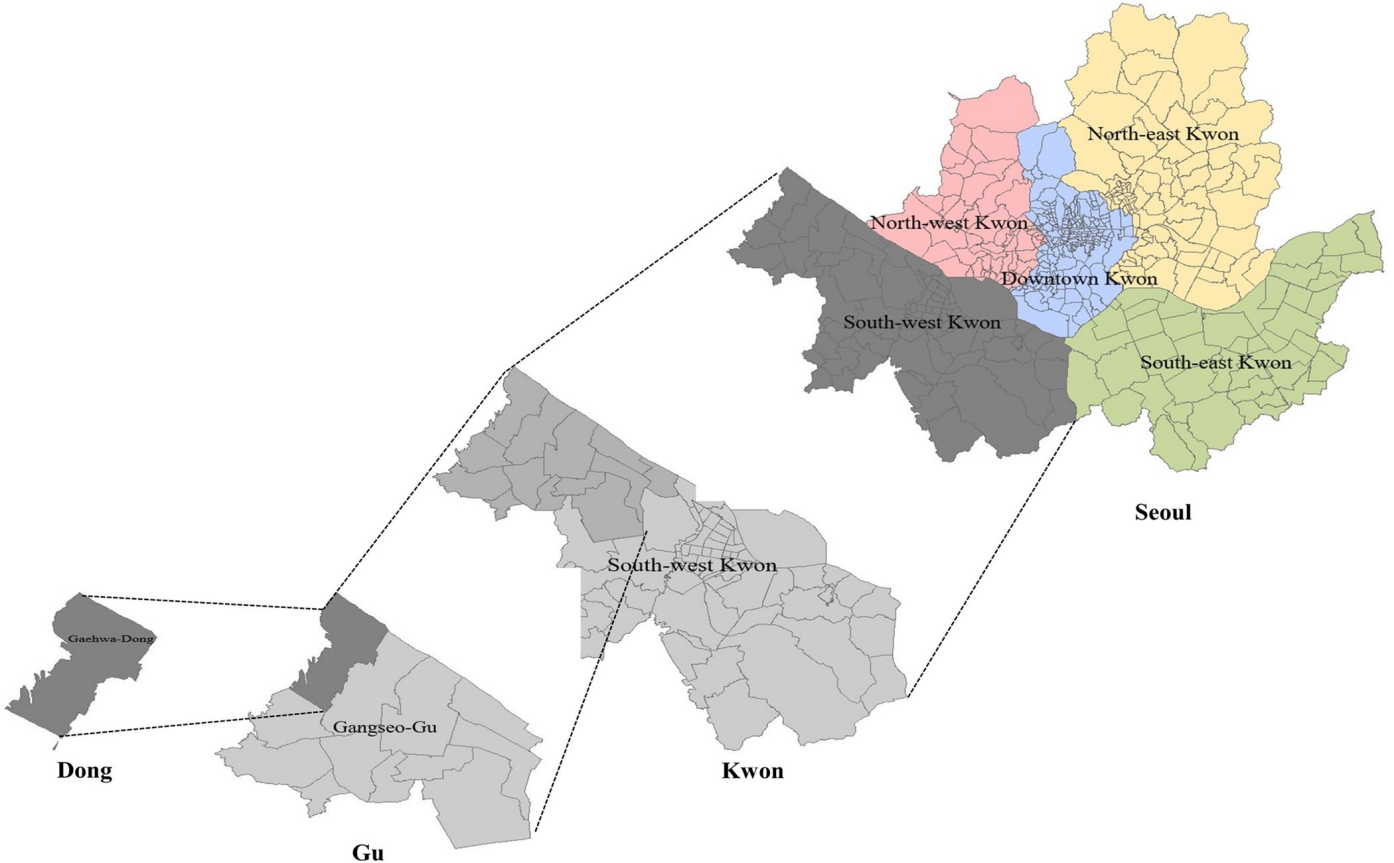
Geography of Seoul City.

Thus, our study aimed to assess 1) the relative importance of two administrative levels, namely “Gu (the second-lowest administrative unit)” and “Dong (the lowest administrative unit)” for geographic variation in the suicide rate at Dong-level, and 2) the proportion of variance in suicide rate at each administrative level explained by Dong-level characteristics in Seoul, the capital city of Korea, based on the most recent dataset of 2014–2016. We additionally performed stratified analyses by another sub-unit of Seoul named “Kwon” which is a level of unit higher than Gu and defined based on residents’ life sphere for the purpose of city development (Fig 1).

## Methods

### Data

#### Unit of analysis

As of 2017, Seoul is divided into 242 Dongs (neighborhood equivalent units), which are nested within 25 Gus (district equivalent units) which are nested again within 5 Kwons (Fig 1). The number of registered populations in each Dong ranged from 164 to 26,594 with the average being 10,245. The Dong was employed as the lowest unit of analysis in this study.

#### Data source

2014–2016 suicide death data for Seoul, aggregated at the Dong-level, were provided by the division of community welfare under the welfare policy office of Seoul city government. Statistics on suicide death in Seoul was originally derived from the nationwide database for cause-specific mortality collected by Statistics Korea. Korea estimates a cause-specific mortality rate classified by the World Health Organization’s International Classification of Disease, based on death reports filed with each administrative office where the deceased resided. The death reports must be supported by a certificate prepared by a doctor to verify death. Although suicide data were collected at the individual level, statistics are only made publicly available for the Gu (for Seoul) or Gun (for other Provinces) level or higher due to the sensitive nature of the topic. Dong-level suicide data is available upon request. The rest of the data was obtained from an online open-access data repository named “Seoul Open Data Plaza” with statistics on a variety of topics about Seoul, including population, household, economy to health, education, and so on. Gu-level suicide rate of Seoul is also available here. Each specific dataset was created by relevant organizations and has different survey intervals and measurement units.

#### Outcome and explanatory variables

The outcome variable is the number of suicide death per 100,000 population by Dong. We performed the indirect age- and sex-standardization of suicide rate using overall age-stratified suicide rate of Seoul population for each year (2014, 2015, and 2016) as the standard population. Explanatory variables included the proportion of males, the poor, the elderly, and the disabled in the population (%), and the number of divorces, entertainment bars, and medical facilities per 100,000 population. These variables were available for all three target years (2014, 2015, and 2016), measured at Dong-level in the data source, and previously found to be associated with suicide rate. The poor was defined as people living below the poverty line (S1 Table). The elderly included people aged more than 65. The disabled were those officially registered on National Pension Service through a screening process. The entertainment bar was defined as a facility that is authorized to provide alcohol, be equipped with entertainment facilities such as karaoke, and employ so-called “hostesses” (paid drinking companions). The density of entertainment bars can be interpreted as a contextual factor considering the documented evidence on the association between alcohol consumption and suicide risk [10,11], and also high risk for suicide attempts among the hostesses due to psychosocial risk factors such as stigma and harassment from employers and clients, economic insecurity, and disrupted social relations due to night work. Finally, the explanatory variable for medical facilities considered all levels of facilities including clinics and hospitals in the main analyses, but we performed sensitivity analyses after the definition of medical facilities to hospital-level only.

#### Analysis

Our analytical data followed the hierarchical structure of Kwon, Gu, and Dong, with the age- and sex-standardized suicide rate per 100,000 persons at the Dong level. The main analyses were performed using a two-level random intercept variance component model with Dong at level 1(i), nested within the Gu at level 2(j). The model takes the following form:

$$y_{ij}=\beta_{0}+\beta_{1}X_{ij}+(u_{0j}+e_{oij})$$

$$\left[u_{0j}]∼N(0,{\sigma_{u0}^{2}}_{}\right)$$

$$\left[e_{0ij}]∼N(0,\sigma_{e0}^{2}\right)$$

The model estimates the standardized suicide rate while adjusting for a vector, *X*_*ij*_, of independent variables measured at Dong-level. Random effects inside the bracket are residual differentials specific for Gu (*u*_0*j*_) and Dong (*e*_*oij*_)-level. Under the i.i.d.(independently and identically distributed) assumption, each set of residuals follows a normal distribution.

We first analyzed a null model with no predictor variables in the fixed part of the model based on the 2014–2016 pooled dataset. We then controlled for the year in model 1. In model 2 ~ 8, we included each of the Dong-level characteristic variables one at a time. Finally, we included all explanatory variables together in model 9. For each model, we calculated the variance partitioning coefficient (VPC), the proportion of variation in suicide rate attributable to Gu and Dong, and the proportional change in variance (PCV) in suicide rate explained by covariates in the subsequent models.

We additionally performed year-stratified analyses and Kwon- stratified analyses using the merged dataset where Dong characteristics were adjusted one at a time in models 1 ~ 7 and all explanatory variables jointly adjusted in model 8 to examine consistency in the fixed effects and the VPC in suicide rate in each administrative level by Kwon and year.

All analyses were performed using Stata 14.0 (StataCorp, College Station, Texas). Map was created using ArcMap 10.7.1 by the author (ESRI, Redlands, California). The shape files of administrative units in Korea were downloaded from GIS DEVELOPER (http://www.gisdeveloper.co.kr/?p=2332) [12].

#### Ethics statement

Data was provided as aggregated at the area-level, not at individual-level. Therefore, no one on the study team had access to identifiers linked to the data. These activities did not meet the regulatory definition of human participant research, and our study was determined to be exempt from a full institutional review.

## Results

Our analytic sample included a total of 424 Dongs nested within 25 Gus from 2014 to 2016 (Table 1). There was an imbalance in the data structure with the smallest Gu (Gangbuk-gu) being comprised of only 13 Dongs and approximately a population size of 0.12 million whereas the largest Gu (Songpa-gu) covered 27 Dongs with around 0.6 million population. The overall age- and sex- standardized suicide rate across all Dongs decreased over time from 24.9 deaths per 100,000 in 2014 to 23.7 deaths in 2016. However, several Gus showed an increasing trend during the study period (e.g, Nowon-gu and Dobong-gu). The mean and the standard deviation (SD) in the suicide rate varied substantially across Gus. In general, Gus with a higher mean suicide rate had larger SDs (corr = 0.67) and the extent of distribution in the suicide rate within Gus was negatively correlated with Dong sample sizes although strength was weak (corr = -0.19).

**Table 1:** Hierarchical structure of area units and standardized suicide rate by Gu and year (per 100,000 persons).

Classification			2014			2015			2016		
Kwon	Gu	Number of Dong	Population	Mean	SD	Population	Mean	SD	Population	Mean	SD
Downtown	Jongno-gu	17	156,993	23.3	12.4	154,986	21.1	10.8	152,737	22.2	13.1
	Jung-gu	15	128,065	25.0	10.7	125,733	25.5	8.3	125,249	21.6	9.7
	Yongsan-gu	16	235,951	28.7	10.9	233,342	28.0	8.8	230,241	29.4	14.3
South-east	Seocho-gu	18	449,678	24.9	11.7	446,764	25.7	15.5	447,192	23.8	8.4
	Gangnam-gu	22	578,114	26.7	13.7	576,495	23.1	7.0	567,115	27.5	12.2
	Songpa-gu	27	664,738	18.8	10.0	660,302	20.7	7.5	657,831	16.2	9.7
	Gangdong-gu	18	476,597	21.9	8.2	458,658	22.2	11.1	444,168	22.5	7.4
North-east	Dongdaemun-gu	14	363,687	24.9	11.0	360,153	23.2	4.9	355,069	25.3	14.8
	Seongdong-gu	17	296,086	24.9	12.5	297,003	25.7	18.2	299,259	22.2	9.1
	Jungnang-gu	16	418,836	24.6	9.7	413,909	25.5	11.5	411,005	21.7	9.2
	Gwangjin-gu	15	363,354	26.6	15.0	360,369	26.7	10.1	357,215	26.0	22.6
	Nowon-gu,	19	582,552	24.0	12.2	574,583	23.0	12.1	567,581	27.0	16.3
	Seongbuk-gu	20	466,706	22.7	9.2	459,275	24.5	11.5	450,355	21.2	7.2
	Gangbuk-gu	13	335,025	19.2	9.8	330,873	21.2	9.6	327,195	16.2	6.2
	Dobong-gu	14	353,709	19.1	11.7	351,242	16.2	9.3	348,220	25.1	13.9
South-west	Dongjak-gu	15	407,470	22.4	9.7	400,641	24.8	12.3	400,997	22.6	9.4
	Gwanak-gu	21	513,186	20.3	9.5	509,663	19.2	8.9	506,851	22.1	12.2
	Guro-gu	15	425,831	26.8	37.8	422,092	28.9	46.4	417,551	26.5	37.2
	Yeongdeungpo-gu	18	382,352	22.6	10.6	378,504	26.4	11.1	370,613	20.8	9.7
	Geumcheon-gu	10	238,463	24.2	12.8	236,284	22.3	14.5	235,386	25.6	13.6
	Yangcheon-gu	18	486,221	23.7	11.1	484,532	21.6	9.6	477,739	21.3	11.0
	Gangseo-gu	20	585,160	23.8	10.7	589,074	22.0	12.5	595,485	24.7	9.9
Norrth-west	Seodaemun-gu	14	310,376	22.3	10.4	312,141	25.0	12.0	314,194	18.4	8.1
	Mapo-gu	16	385,439	37.4	31.9	387,643	37.1	36.0	379,892	36.7	31.1
	Eunpyeong-gu	16	498,644	28.4	13.5	497,920	24.9	13.5	491,476	26.1	10.4
Total	25	424	10,103,233	24.9	14.3	10,022,181	24.2	17.3	9,930,616	23.7	15.7

SD: Standard Deviation.

**Fig 2:**
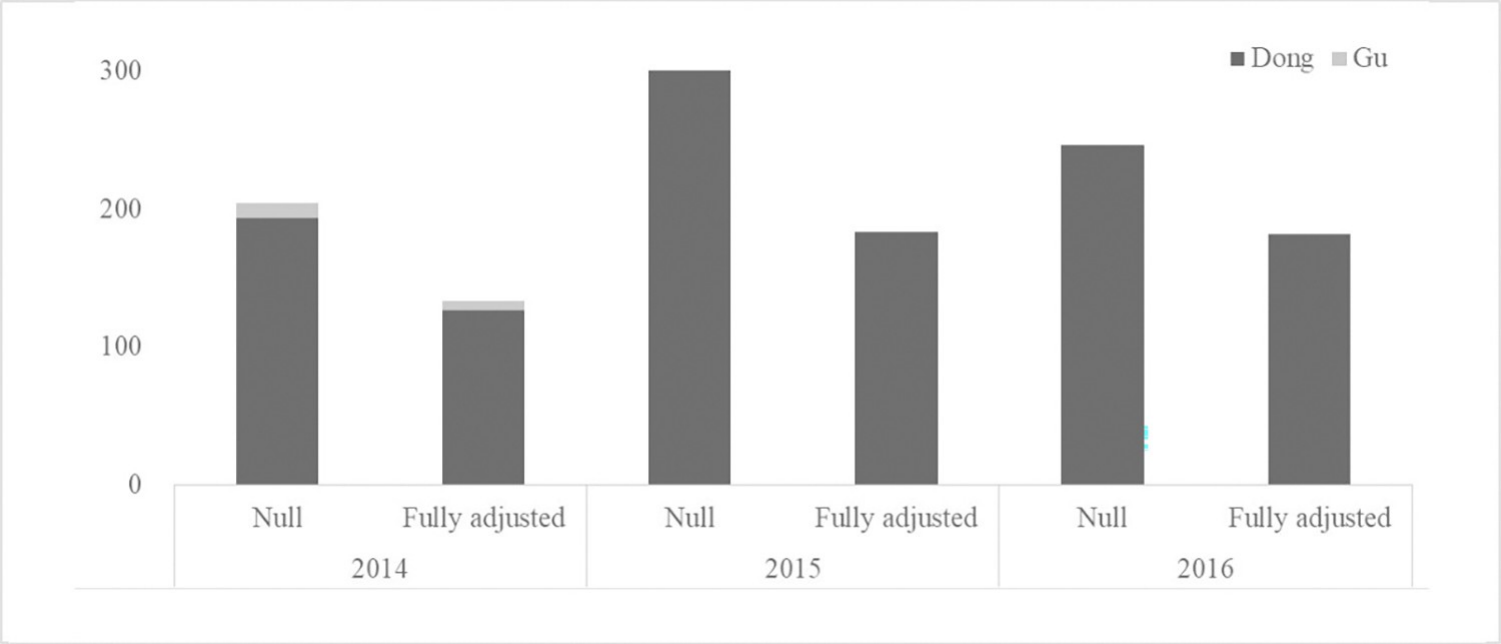
Variance estimates in null and fully adjusted two-level random intercept models for suicide rate, stratified by year.

Of the total variance (var_Gu+Dong_) in suicide rate estimated from the null model using the pooled data, 96.6% was attributable to the within-Gu, between-Dong differences while the remaining 3.4% was attributed to the between-Gu differences (Table 2). In year-stratified analyses, the total variance in suicide rate estimated from the null model was 204.5 in 2014, 300.1 in 2015, and 246.5 in 2016 (Fig 2) (S2 Table), of which 94.7%, 100%, and 99.9% was attributable to the within-Gu, between-Dong differences in each year respectively, indicating the small and decreased contribution of the Gu- level during the study period. The between-Dong variation in suicide rate decreased in absolute terms after adjusting for the Dong-level characteristics but still accounted for most of the variation in suicide rate in both merged and year-stratified analyses (Fig 2) (Tables 2 and S2).

**Table 2:** Bivariate and fully- adjusted association between independent variables and standardized suicide rate based on merged dataset 2014~2016.

	Variable		Null		M1		M2		M3		M4		M5		M6		M7		M8		M9	
			b	*P*	b	*P*	b	*P*	b	*P*	b	*P*	b	*P*	b	*P*	b	*P*	b	*P*	b	*P*
Fixed part	Year				-0.59	0.266	-0.41	0.43	-0.59	0.265	-0.59	0.268	-0.56	0.295	-0.09	0.85	-0.57	0.274	-0.48	0.383	-0.03	0.954
	%, the male						1.82	<0.001													0.28	0.262
	%, the poor								0.82	<0.001											2.08	<0.001
	%, the elderly										0.71	<0.001									0.22	0.252
	%, the disabled												0.51	0.05							-3.09	<0.001
	N of divorces per 100,000														0.05	<0.001					0.05	<0.001
	N of bars per 100,000																0.02	<0.001			0.01	0.017
	N of medical facilities per 100,000																		0.02	<0.001	0.01	<0.001
Random Part	Gu	Nu	25		25		25		25		25		25		25		25		25		25	
		Variance (SE)	8.4(3.9)		8.4(3.9)		5.2(2.9)		7.0(3.4)		6.7(3.3)		7.5(3.6)		5.4(2.7)		3.0(2.1)		4.5(2.6)		2.2(1.7)	
		VPC (%)	3.4		3.4		2.2		2.8		2.7		3.0		2.8		1.3		1.9		1.3	
		PCV (%) vs. M1	-		-		38.0		17.2		20.6		11.6		35.7		64.9		46.3		73.5	
	Dong	N	1,271		1,271		1,271		1,271		1,271		1,271		1,271		1,271		1,181		1,181	
		Variance (SE)	242.5(9.7)		242.3(9.7)		233.4(9.4)		240.1(9.6)		239.8(9.6)		241.9(9.7)		191.5(7.7)		227.3(9.1)		228.6(9.5)		169.5(7.1)	
		VPC (%)	96.6		96.6		97.8		97.2		97.3		97.0		97.2		98.7		98.1		98.7	
		PCV (%) vs. M1	-		-		3.7		0.9		1.1		0.1		21.0		6.2		5.6		30.1	

1) M: Model, SE: Standard Error, VPC: Variance partition coefficient, PCV: Proportional change in variance.

In the pooled data adjusting for year fixed effects, all the Dong-level characteristics were significantly associated with suicide rate when individually considered (M2~M8 in Table 2). When all variables were simultaneously considered in the fully adjusted model, the proportion of the poor (b = 2.08) and the disabled (b = -3.09), the number of divorce cases (b = 0.05), entertainment bars (b = 0.01), and medical facilities (b = 0.01) per 100,000 population remained statistically significant (p<0.001 for all) (M9 in Table 2). When stratified by year, the bivariate association between each of the Dong-level characteristics and suicide rate was found to be highly consistent over time (S2 Table). In the year-specific fully adjusted models, only the number of divorce cases per 100,000 population was significant across all three years (b = 0.05 ~ 0.06, p<0.001 for all three years). The proportion of the disabled was negatively associated with the suicide rate in 2015 and 2016 (b = -3.13, p<0.001 in 2015 and b = -3.88, p<0.001 in 2016).

In pooled analyses, most of the variance was concentrated at the Dong-level (VPC_Gu_ = 3.4%; VPC_Dong_ = 96.6% in the null model) (Table 2). At the Gu-level, the proportion of variance explained ranged substantially depending on Dong-level characteristics. 11.6% of between-Gu differences were explained by the proportion of the disabled population while as high as 64.9% of the variation was explained by the density of the bars when compared to variance in model 1 (only year-adjusted). The biggest percent of the between-Dong differences in suicide rate was explained by the number of divorce cases per 100,000 (21.0%). Other variables explained less than 10% of the between-Dong variation in the suicide rate. Adjusting for all the Dong-level characteristics explained a total of 73.5% of the between-Gu variation and 30.1% of the between-Dong variation in the suicide rate (M9 in Table 2). The contribution of these Dong-level characteristics in explaining variation at Dong-level slightly increased from 34.7% in 2014 to 38.9% in 2015 but decreased to 26.3% in 2016 (S2 Table).

**Fig 3:**
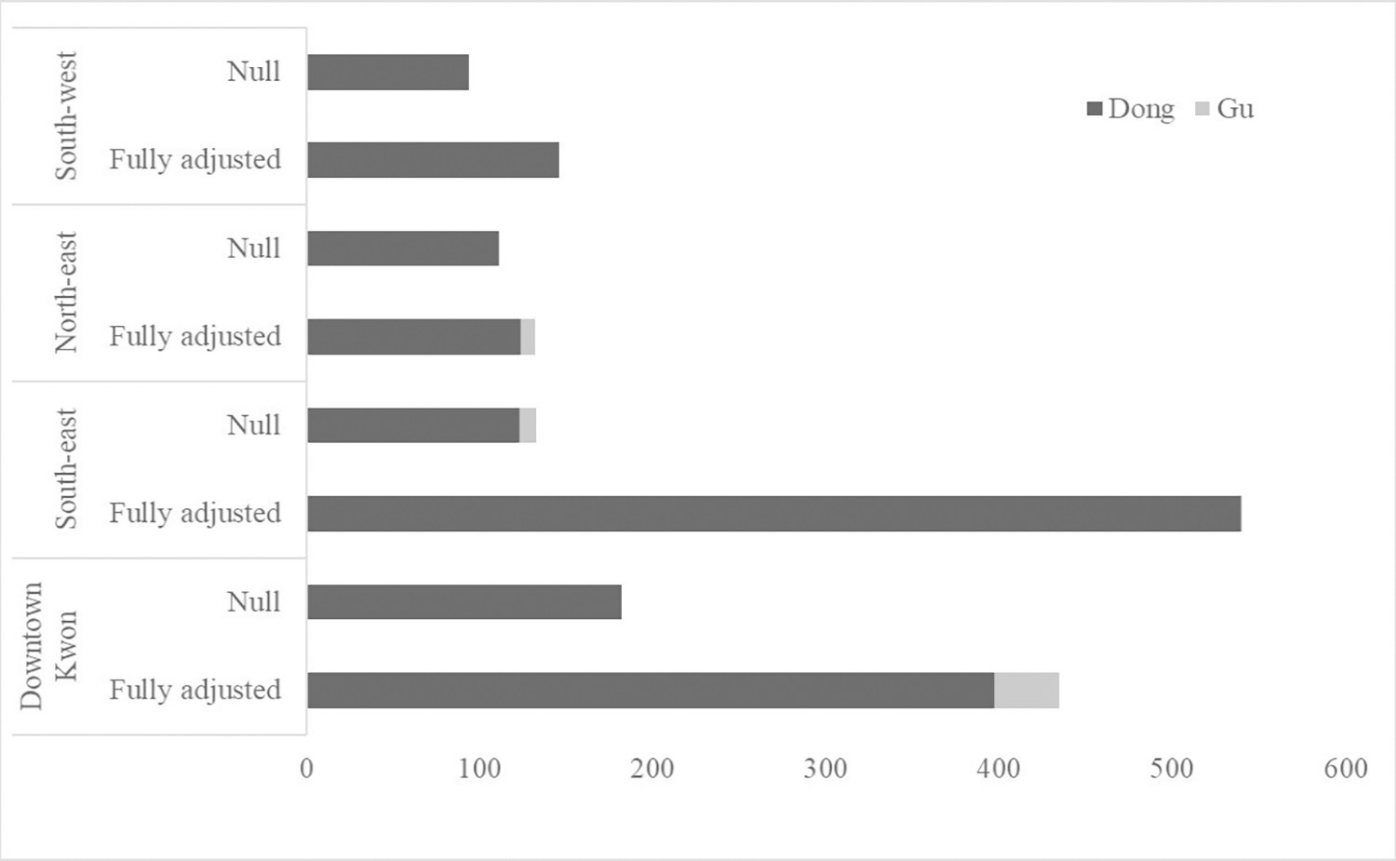
Variance estimates in null and fully adjusted two-level random intercept models for suicide rate, stratified by Kwon.

In Kwon specific analysis, the total variation in suicide rate in Southeast Kwon (var_Gu+Dong_ = 539.6), when adjusting for year-fixed effect only, was about 4.9 times larger than that in Northwest Kwon (var_Gu+Dong_ = 110.0) (Fig 3) (S3 Table). Across all Kwons, 91.3 ~ 100% of the total variation in suicide rate was consistently at the Dong-level. A relatively larger between-Gu variation was found within the Downtown Kwon (var_Gu_ = 37.9; VPC_Gu_ = 8.7%) and Northeast Kwon (var_Gu_ = 8.6; VPC_Gu_ = 6.5%) whereas it was less than 1% in other Kwons. After adjusting for all the Dong-level characteristics, the total variation in the suicide rate was the least for Northwest (var_Gu+Dong_ = 93.9) and was the largest for the Downtown Kwon (var_Gu+Dong_ = 224.4).

### Sensitivity analysis

A sensitivity analysis was conducted after limiting the definition of medical facilities to hospital-level only (i.e, excluding smaller clinics, nursing facilities, etc). Compared to the results in the main analysis, the average association between the density of medical facilities and the suicide rate was stronger in both bivariate (results not shown) and fully adjusted model (b = 0.39, p = <0.001 vs. b = 0.01, p = <0.001 for all types of medical facilities) in the sensitivity analysis (S4 Table). The proportion of variation attributable to Gu and Dong were similar to the model when all types of medical facilities were included (S4 Table).

## Discussion

Although the importance of area-specific approaches to suicide prevention has been increasingly emphasized in Korea, none of the prior studies have considered which level of geography or administrative level is relatively more important in shaping the distribution of suicide rate nor how much of variance in suicide rate can be explained by Dong-level risk factors. A few salient findings from our multilevel analysis of Seoul’s suicide data from 2014 to 2016 are worth highlighting.

First, when Dong and Gu units were simultaneously considered, most of the variation in suicide rate was attributed to within-Gu, between-Dong differences with a contribution of Gu-level being small and decreasing over time. This result is in line with many previous articles that have emphasized the importance of local neighborhood context on health outcomes [13]. In a geographic sense, “neighborhood” has been defined in diverse ways, but it typically refers to a spatial area smaller than a municipality but larger than a block with approximately 5,000 to 10,000 residents and with relatively homogenous socioeconomic characteristics. Throughout the literature, neighborhood has been interpreted more as an ecological unit rather than a geographical unit shaping the behavior of its members, resulting in inter-dependence among its inhabitants. In Korea, a unit which can be considered equivalent to a neighborhood is Dong both in a geographical and ecological sense [14–16]. Gu, on the other hand, as a primary local government, functions more as a geographical delineation for administrative purposes rather than an ecological unit although there may be some contextual effects at the Gu-level, such as financing capacity for welfare provision, as a results of autonomous local government system, which was introduced in 1991 [17,18]. Much larger variation between Dong units as opposed to Gu units in suicide rate indicate that causes for suicide rate are more likely to operate at Dong or individual-levels rather than at the Gu-level. As specified in the 2018 national strategy for suicide prevention, the Korean government is implementing interventions at a various administrative level, such as placing suicide gatekeepers at every Dong (those specially trained for detecting people at higher risk for suicide in the community and referring them to a counselor) and operating mental health welfare centers at every Gu, but with greater emphasis on Gu or Kwon rather than Dong. Our findings provide supporting evidence that more attention needs to be paid to smaller units.

Second, Dong-level characteristics in our analyses jointly explained more than 73.5% of the between-Gu variation and up to 30.1% of the between-Dong variation in suicide rate in Seoul. The number of divorce cases per 100,000 consistently explained the largest fraction of variation at the Dong-level. Divorce has been consistently observed as a risk factor for suicide in previous studies both domestically [19,20] and globally [21,22]. First, divorce disrupts family and social ties, reducing individual integration into society and causing psychological distress [4,23–25]. Second, emotional interdependence between married couples is lost after divorce raising emotional distress [24,26]. Third, the divorced, especially women, are likely to face financial plight from inadequate welfare support or single parenting burden [24,27,28]. All these factors could theoretically contribute to the compositional effect of higher suicide rate among the divorced.

A few unexpected findings were observed in our study. The association between the density of medical facilities and suicide rate was positive in the pooled analyses, which became even more pronounced when we restricted the definition of the medical facility only to hospital-level in the sensitivity analysis. Our initial hypothesis was that the density of medical facilities may reflect greater access to health care, increasing the likelihood of people at higher risk of intentional self-harm to get detected and receive appropriate treatment earlier, and also for people who attempt suicide to be sent to the emergency department promptly, thereby leading to decreased suicide rate. However, high density of medical facilities may also indicate a high demand for medical service, which is a proxy for higher prevalence of illnesses in the area (endogeneity). This might explain the unexpected positive association between the density of medical facilities and suicide rate at Dong-level. Another unexpected finding was that the association between the proportion of the disabled and suicide rate changed from being positive in bivariate analyses to negative in fully-adjusted analyses. Although empirical evidence linking disability and completed suicide remains sparse, it is well documented that disability is positively associated with higher suicide ideation due to psychological difficulties resulting from discrimination and social exclusion, as well as financial constraints [29]. We added other covariates alternately to the bivariate model to detect when the direction of the coefficient is flipped. The positive bivariate association between the proportion of the disabled and suicide rate turned opposite when we included the proportion of the poor and the elderly, which can be interpreted as most of the suicides among the disabled occurring among the elderly and the poor.

Third, stratified analysis by Kwon indicated a disproportionately large variation in suicide rate at the Southeast Kwon compared to other Kwons. Gangnam-gu, Seocho-gu, and Songpa-gu in Southeast Kwon are the top three in terms of wealth level in Seoul as well as nationwide. Ironically, at another extreme within these Gus, informal settlers who are the poorest of the poor are residing. Also, adult entertainment business zones, around which many female sex workers reside alone, are concentrated in a few Dongs of these Gus. Co-residing of the highest and the lowest risk groups in the same Kwon may explain the disproportionately large variation observed in Southeast Kwon.

Our study has a few important limitations to consider when interpreting the findings. First, as individual-level suicide data are not accessible, the lowest-level unit of analysis in our study was Dong. This means that variance at the individual-level is currently captured in the Dong-level variation and cannot be separated out, which precludes controlling for possible compositional confounding. Although it is not likely that Gu-level variation is greater than Dong-level variation even if individual-level are considered together, the between-Dong variation in our study needs to be interpreted with caution. Second, although we aggregated the data across a period of three years, which is the maximum number of years available, to ensure sufficient incidence at the Dong-level, it’s possible that it was not enough to produce stable estimates. However, it should be kept in mind, too, that aggregating the long-term years also would have the disadvantage of hiding different secular trends in suicide between Dongs. Third, the lack of data prevented comprehensive control for Dong-level characteristics. For instance, information on education level and health status at Dong-level was not available although they are known to be significant determinants of suicide [30]. Diverse Dong-level variables need to be measured and made available for future research. Fourth, the suicide rate at area-level is determined by the residency of the deceased since the death report is filed with the administrative office in the area where the deceased resided. Although we assume that the context of their habitation had a greater impact on their suicide than the context of the place where they committed suicide, we cannot rule out the possibility that they were influenced by the factors outside their residential area. Interventions planned by evidence based on the administrative unit of domicile would be inappropriate in this circumstance. Lastly, due to the lack of national data on suicide rate disaggregated at the Dong-level, our study was restricted to Seoul. However, evidence that aids the reduction of suicide in Seoul city is expected to ultimately contribute to suicide reduction at the national level. Furthermore, the multi-level approaches taken here can apply to other health outcomes and other contexts.

## Conclusion

To our knowledge, we present the first quantitative evidence emphasizing the importance of micro-level ecological area unit, Dong, in reducing the geographic variation in suicide rate in Seoul. Our study provides relevant references for suicide interventions in other countries. Governments need to prioritize identifying the ecological unit that generates the biggest variance in the suicide rate in the context of their own country and design the policy targeted to those specific units rather than practicing conventional administrative unit-based policy. For this, disaggregated data at the micro-level are required for more precise, targeted planning and implementation of area-based suicide prevention policies and programs.

## Supporting information

S1 Table Poverty line in 2014, 2015, and 2016. (DOCX)

S2 Table Year-stratified bivariate and fully- adjusted association between independent variables and standardized suicide rate. (DOCX)

S3 Table Kwon-stratified bivariate and fully- adjusted regression based on merged dataset. (DOCX)

S4 Table Bivariate and fully- adjusted regression when adjusted for the number of hospital-level medical institutions. (DOCX)
