# Supplementary material for: The relative importance of macro versus micro geographical scale in explaining suicide variation in Seoul, South Korea 2014–2016
Source: PLoS One. 2022 Sep 9;17(9):e0273866. doi: 10.1371/journal.pone.0273866 (PMC9462743; doi:10.1371/journal.pone.0273866)
Supplement: S1 Table — (DOCX) [file pone.0273866.s001.docx]

Table S1. Poverty line in 2014, 2015, and 2016.

| Number of household members | 2014 | | 2015 | | 2016 | |
| --- | --- | --- | --- | --- | --- | --- |
|  | Korean Won | US$ | Korean Won | US$ | Korean Won | US$ |
| 1 | 603,403 | 563 | 617,281 | 536 | 649,932 | 550 |
| 2 | 1,027,417 | 959 | 1,051,048 | 912 | 1,106,642 | 937 |
| 3 | 1,329,118 | 1,240 | 1,359,688 | 1,180 | 1,431,608 | 1,212 |
| 4 | 1,630,820 | 1,522 | 1,688,329 | 1,466 | 1,756,574 | 1,487 |
| 5 | 1,932,522 | 1,803 | 1,976,970 | 1,716 | 2,081,540 | 1,762 |
| 6 | 2,234,223 | 2,085 | 2,285,610 | 1,984 | 2,406,506 | 2,037 |
| 7 | 2,535,925 | 2,366 | 2,594,251 | 2,252 | 2,731,473 | 2,312 |
| 8 | 2,837,627 | 2,648 | 2,902,892 | 2,520 | 3,056,440 | 2,587 |
| 9 | 3,139,329 | 2,929 | 3,211,533 | 2,788 | 3,381,407 | 2,862 |
| 10 | 3,441,031 | 3,211 | 3,520,174 | 3,056 | 3,706,374 | 3,137 |
| 11 | 3,742,733 | 3,492 | 3,828,815 | 3,324 | 4,031,341 | 3,412 |
