# Supplementary material for: The relative importance of macro versus micro geographical scale in explaining suicide variation in Seoul, South Korea 2014–2016
Source: PLoS One. 2022 Sep 9;17(9):e0273866. doi: 10.1371/journal.pone.0273866 (PMC9462743; doi:10.1371/journal.pone.0273866)
Supplement: S2 Table — (DOCX) [file pone.0273866.s002.docx]

Table S2. Year-stratified bivariate and fully- adjusted association between independent variables and standardized suicide rate.

|  | **Variable** | | Null | | M1 | | M2 | | M3 | | M4 | | M5 | | M6 | | M7 | | M8 | | |
| --- | --- | --- | --- | --- | --- | --- | --- | --- | --- | --- | --- | --- | --- | --- | --- | --- | --- | --- | --- | --- | --- |
|  |  |  | b | *P* | b | *P* | b | *P* | b | *P* | b | *P* | b | *P* | b | *P* | b | *P* | b | *P* |  |
| 2014 |  | |  |  |  |  |  |  |  |  |  |  |  |  |  |  |  |  |  |  |  |
| Fixed part | % the male | |  |  | 1.84 | <0.001 |  |  |  |  |  |  |  |  |  |  |  |  | 0.44 | 0.277 |  |
|  | % the poor | |  |  |  |  | 0.86 | 0.009 |  |  |  |  |  |  |  |  |  |  | 0.76 | 0.216 |  |
|  | % the elderly | |  |  |  |  |  |  | 0.56 | 0.031 |  |  |  |  |  |  |  |  | -0.19 | 0.555 |  |
|  | % the disabled | |  |  |  |  |  |  |  |  | 0.48 | 0.218 |  |  |  |  |  |  | -1.11 | 0.206 |  |
|  | N of divorces per 100,000 | |  |  |  |  |  |  |  |  |  |  | 0.04 | <0.001 |  |  |  |  | 0.05 | <0.001 |  |
|  | N of bars** per 100,000 | |  |  |  |  |  |  |  |  |  |  |  |  | 0.02 | <0.001 |  |  | 0.01 | 0.071 |  |
|  | N of medical facilities per 100,000 | |  |  |  |  |  |  |  |  |  |  |  |  |  |  | 0.01 | <0.001 | 0.00 | 0.227 |  |
| Random Part | Gu | N | 25 | | 25 | | 25 | | 25 | | 25 | | 25 | | 25 | | 21 | | 21 | | |
|  |  | Variance (SE) | 10.9(6.5) | | 7.0(5.3) | | 8.1(5.7) | | 8.5(5.8) | | 9.3(6.2) | | 8.0(4.7) | | 5.5(4.7) | | 8.1(6.3) | | 7.1(4.7) | | |
|  |  | VPC (%) | 5.3 | | 3.6 | | 4.0 | | 4.2 | | 4.6 | | 5.2 | | 2.9 | | 4.0 | | 5.3 | | |
|  |  | PCV (%) vs. M1 | - | | 35.7 | | 26.0 | | 21.9 | | 14.8 | | 27.0 | | 49.9 | | 25.7 | | 34.9 | | |
|  | Dong | N | 423 | | 423 | | 423 | | 423 | | 423 | | 423 | | 423 | | 353 | | 353 | | |
|  |  | Variance (SE) | 193.6(13.7) | | 185.5(13.2) | | 192.1(13.6) | | 192.9(13.7) | | 193.8(13.8) | | 144.1(10.2) | | 181.6(12.9) | | 196.4(15.2) | | 126.5(9.8) | | |
|  |  | VPC (%) | 94.7 | | 96.4 | | 96.0 | | 95.8 | | 95.4 | | 94.8 | | 97.1 | | 96.0 | | 94.7 | | |
|  |  | PCV (%) vs. M1 | - | | 4.2 | | 0.8 | | 0.4 | | -0.1 | | 25.6 | | 6.2 | | -1.4 | | 34.7 | | |
| 2015 |  | |  |  |  |  |  |  |  |  |  |  |  |  |  |  |  |  |  |  |  |
| Fixed part | % the male | |  |  | 1.68 | <0.001 |  |  |  |  |  |  |  |  |  |  |  |  | -0.14 | 0.745 |  |
|  | % the poor | |  |  |  |  | 1.04 | 0.008 |  |  |  |  |  |  |  |  |  |  | 2.55 | <0.001 |  |
|  | % the elderly | |  |  |  |  |  |  | 0.91 | 0.002 |  |  |  |  |  |  |  |  | 0.21 | 0.488 |  |
|  | % the disabled | |  |  |  |  |  |  |  |  | 0.89 | 0.06 |  |  |  |  |  |  | -3.13 | <0.001 |  |
|  | N of divorces per 100/000 | |  |  |  |  |  |  |  |  |  |  | 0.05 | <0.001 |  |  |  |  | 0.06 | <0.001 |  |
|  | N of bars** per 100/000 | |  |  |  |  |  |  |  |  |  |  |  |  | 0.03 | <0.001 |  |  | 0.00 | 0.429 |  |
|  | N of medical facilities per 100/000 | |  |  |  |  |  |  |  |  |  |  |  |  |  |  | 0.02 | <0.001 | 0.01 | <0.001 |  |
| Random part | Gu | N | 25 | | 25 | | 25 | | 25 | | 25 | | 25 | | 25 | | 24 | | 24 | | |
|  |  | Variance (SE) | 0(0) | | 0(0) | | 0 (0) | | 0 (0) | | 0 (0) | | 0(0) | | 0(0) | | 0 (0) | | 0(0) | | |
|  |  | VPC (%) | 0 | | 0 | | 0 | | 0 | | 0 | | 0 | | 0 | | 0 | | 0 | | |
|  |  | PCV (%) vs. M1 | N/A | | N/A | | N/A | | N/A | | N/A | | N/A | | N/A | | N/A | | N/A | | |
|  | Dong | N | 424 | | 424 | | 424 | | 424 | | 424 | | 424 | | 424 | | 404 | | 404 | | |
|  |  | Variance (SE) | 300.1(20.6) | | 290.2(19.9) | | 295.2(20.3) | | 293.7(20.2) | | 297.6(20.4) | | 224.1(15.4) | | 277.8 (19.1) | | 251.9 (16.1) | | 183.4(12.9) | | |
|  |  | VPC (%) | 100 | | 100 | | 100 | | 100 | | 100 | | 100 | | 100 | | 100 | | 100 | | |
|  |  | PCV (%) vs. M1 | - | | 3.3 | | 1.7 | | 2.1 | | 0.8 | | 25.3 | | 7.4 | | 16.1 | | 38.9 | | |
| 2016 |  | |  |  |  |  |  |  |  |  |  |  |  |  |  |  |  |  |  |  |  |
| Fixed part | % the male | |  |  | 1.98 | <0.001 |  |  |  |  |  |  |  |  |  |  |  |  | 0.60 | 0.14 |  |
|  | % the poor | |  |  |  |  | 0.61 | 0.08 |  |  |  |  |  |  |  |  |  |  | 2.32 | <0.001 |  |
|  | % the elderly | |  |  |  |  |  |  | 0.61 | 0.03 |  |  |  |  |  |  |  |  | 0.29 | 0.331 |  |
|  | % the disabled | |  |  |  |  |  |  |  |  | 0.35 | 0.43 |  |  |  |  |  |  | -3.88 | <0.001 |  |
|  | N of divorces per 100,000 | |  |  |  |  |  |  |  |  |  |  | 0.04 | <0.001 |  |  |  |  | 0.05 | <0.001 |  |
|  | N of bars per 100,000 | |  |  |  |  |  |  |  |  |  |  |  |  | 0.03 | <0.001 |  |  | 0.01 | 0.025 |  |
|  | N of medical facilities per 100,000 | |  |  |  |  |  |  |  |  |  |  |  |  |  |  | 0.01 | <0.001 | 0.00 | 0.167 |  |
| Random part | Gu | N | 25 | | 25 | | 25 | | 25 | | 25 | | 25 | | 25 | | 25 | | 25 | | |
|  |  | Variance (SE) | 0.3(4.6) | | 0(0) | | 0(0) | | 0.5(4.3) | | 0(0) | | 0(0) | | 0.3(4.1) | | 0.4(4.3) | | 0(0) | | |
|  |  | VPC (%) | 0.1 | | 0 | | 0 | | 4.3 | | 0 | | 0 | | 0.1 | | 0.2 | | 0 | | |
|  |  | PCV (%) vs. M1 | - | | N/A | | N/A | | N/A | | N/A | | N/A | | N/A | | N/A | | N/A | | |
|  | Dong | N | 424 | | 424 | | 424 | | 424 | | 424 | | 424 | | 424 | | 424 | | 424 | | |
|  |  | Variance (SE) | 246.3(17.5) | | 232.6(16.0) | | 244.8(16.8) | | 243.7(16.7) | | 246.2(16.9) | | 209.7(14.9) | | 225.6(15.5) | | 230.9(15.9) | | 181.5(12.5) | | |
|  |  | VPC (%) | 99.9 | | 100 | | 100 | | 100 | | 100 | | 99.4 | | 100 | | 100 | | 100 | | |
|  |  | PCV (%) vs. M1 | - | | 5.6 | | 0.6 | | 1.1 | | 0 | | 14.8 | | 8.4 | | 6.2 | | 26.3 | | |

1) M: Model, SE: Standard Error, VPC: Variance partition coefficient, PCV: Proportional change in variance, N/A: Non Available.
