# Supplementary material for: The relative importance of macro versus micro geographical scale in explaining suicide variation in Seoul, South Korea 2014–2016
Source: PLoS One. 2022 Sep 9;17(9):e0273866. doi: 10.1371/journal.pone.0273866 (PMC9462743; doi:10.1371/journal.pone.0273866)
Supplement: S3 Table — (DOCX) [file pone.0273866.s003.docx]

Table S3. Kwon-stratified bivariate and fully- adjusted regression based on merged dataset.

|  | **Variable** | | Null | | M1 | | M2 | | M3 | | M4 | | M5 | | M6 | | M7 | | M8 | | M9 | |
| --- | --- | --- | --- | --- | --- | --- | --- | --- | --- | --- | --- | --- | --- | --- | --- | --- | --- | --- | --- | --- | --- | --- |
|  |  | | b | *P* | b | *P* | b | *P* | b | *P* | b | *P* | b | *P* | b | *P* | b | *P* | b | *P* | b | *P* |
| Downtown Kown | | |  |  |  |  |  |  |  |  |  |  |  |  |  |  |  |  |  |  |  |  |
| Fixed  part | year | |  |  | -2.29 | 0.257 | -2.02 | 0.29 | -2.29 | 0.257 | -2.29 | 0.257 | -2.66 | 0.179 | -1.82 | 0.367 | -2.14 | 0.223 | -2.27 | 0.199 | -2.02 | 0.194 |
|  | % the male | |  |  |  |  | 2.04 | <0.001 |  |  |  |  |  |  |  |  |  |  |  |  | 4.04 | <0.001 |
|  | % the poor | |  |  |  |  |  |  | -0.02 | 0.976 |  |  |  |  |  |  |  |  |  |  | -3.31 | 0.035 |
|  | % the elderly | |  |  |  |  |  |  |  |  | 0.16 | 0.79 |  |  |  |  |  |  |  |  | -1.17 | 0.068 |
|  | % the disabled | |  |  |  |  |  |  |  |  |  |  | -3.78 | 0.015 |  |  |  |  |  |  | -2.82 | 0.145 |
|  | N of divorces per 100,000 | |  |  |  |  |  |  |  |  |  |  |  |  | 0.02 | 0.048 |  |  |  |  | 0.00 | 0.764 |
|  | N of bars per 100,000 | |  |  |  |  |  |  |  |  |  |  |  |  |  |  | 0.02 | <0.001 |  |  | -0.01 | 0.396 |
|  | N of medical facilities per 100,000 | |  |  |  |  |  |  |  |  |  |  |  |  |  |  |  |  | 0.01 | <0.001 | 0.01 | <0.001 |
| Random  Part | Gu | N | 3 | | 3 | | 3 | | 3 | | 3 | | 3 | | 3 | | 3 | | 3 | | 3 | |
|  |  | Variance (SE) | 37.9(37.9) | | 38.0(37.9) | | 21.8(24.2) | | 38.1(38.0) | | 37.7(37.7) | | 49.4(47.2) | | 31.8(32.8) | | 6.4(10.5) | | 6.3(10.6) | | 2.8(7.1) | |
|  |  | VPC (%) | 8.7 | | 8.8 | | 5.9 | | 8.8 | | 8.8 | | 11.6 | | 7.6 | | 2.1 | | 2.1 | | 1.2 | |
|  |  | PCV (%) vs. M1 | - | | - | | 42.7 | | -0.1 | | 0.7 | | -30.0 | | 16.4 | | 83.3 | | 83.3 | | 92.7 | |
|  | Dong | N | 144 | | 144 | | 144 | | 144 | | 144 | | 144 | | 144 | | 144 | | 144 | | 141 | |
|  |  | Variance (SE) | 396.8(47.3) | | 393.2(46.8) | | 350.0(41.7) | | 393.2(46.8) | | 393.1(46.8) | | 375.9(44.8) | | 383.9(45.7) | | 296.3(35.3) | | 295.4(35.6) | | 221.6(26.8) | |
|  |  | VPC (%) | 91.3 | | 91.2 | | 94.1 | | 91.2 | | 91.2 | | 88.4 | | 92.4 | | 97.9 | | 97.9 | | 98.8 | |
|  |  | PCV (%) vs. M1 | - | | - | | 11.0 | | 0.0 | | 0.0 | | 4.4 | | 2.4 | | 24.6 | | 24.9 | | 43.6 | |
| Southeast Kown | | |  |  |  |  |  |  |  |  |  |  |  |  |  |  |  |  |  |  |  |  |
| Fixed part | year | |  |  | -0.58 | 0.746 | -0.35 | 0.84 | -0.57 | 0.75 | -0.55 | 0.757 | -0.55 | 0.759 | 0.21 | 0.882 | -0.59 | 0.736 | -0.79 | 0.672 | 0.67 | 0.544 |
|  | % the male | |  |  |  |  | 4.16 | <0.001 |  |  |  |  |  |  |  |  |  |  |  |  | 2.89 | <0.001 |
|  | % the poor | |  |  |  |  |  |  | 0.51 | 0.432 |  |  |  |  |  |  |  |  |  |  | 5.63 | <0.001 |
|  | % the elderly | |  |  |  |  |  |  |  |  | 0.81 | 0.18 |  |  |  |  |  |  |  |  | 0.46 | 0.341 |
|  | % the disabled | |  |  |  |  |  |  |  |  |  |  | 0.20 | 0.712 |  |  |  |  |  |  | -7.40 | <0.001 |
|  | N of divorces per 100,000 | |  |  |  |  |  |  |  |  |  |  |  |  | 0.05 | <0.001 |  |  |  |  | 0.08 | <0.001 |
|  | N of bars per 100,000 | |  |  |  |  |  |  |  |  |  |  |  |  |  |  | 0.10 | <0.001 |  |  | 0.0 | 0.6 |
|  | N of medical facilities per 100,000 | |  |  |  |  |  |  |  |  |  |  |  |  |  |  |  |  | 0.03 | <0.001 | 0.0 | <0.001 |
| Random  Part | Gu | N | 4 | | 4 | | 4 | | 4 | | 4 | | 4 | | 4 | | 4 | | 4 | | 4 | |
|  |  | Variance (SE) | 0.4(7.5) | | 0.6(7.7) | | 0.6(7.0) | | 0.4(7.1) | | 0.8(7.5) | | 0.2(7.3) | | 0.0(0.0) | | 3.4 (8.5) | | 12.9(14.7) | | 0 (0) | |
|  |  | VPC (%) | 0.1 | | 0.1 | | 0.1 | | 0.1 | | 0.1 | | 0 | | 0 | | 0.7 | | 2.6 | | 0 | |
|  |  | PCV (%)^*^ | - | | - | | 4.8 | | 28.9 | | -20.1 | | 63.4 | | 100 | | -438.7 | | -1934.4 | | 100 | |
|  | Dong | N | 255 | | 255 | | 254 | | 255 | | 255 | | 254 | | 254 | | 254 | | 235 | | 235 | |
|  |  | Variance (SE) | 539.2(48.4) | | 538.8(48.3) | | 508.8(45.5) | | 537.7(48.2) | | 534.9(48.0) | | 538.9(48.4) | | 328.0(29.1) | | 507.3(45.4) | | 482.1(44.9) | | 182.1(16.8) | |
|  |  | VPC (%) | 99.9 | | 99.9 | | 99.9 | | 99.9 | | 99.9 | | 100 | | 100 | | 99.3 | | 97.4 | | 100 | |
|  |  | PCV (%)^*^ | - | | - | | 5.7 | | 0.2 | | 0.7 | | 0.0 | | 39.1 | | 5.8 | | 10.5 | | 66.2 | |
| Northeast Kown | | |  |  |  |  |  |  |  |  |  |  |  |  |  |  |  |  |  |  |  |  |
| Fixed  part | year | |  |  | -0.89 | 0.209 | -0.77 | 0.276 | -0.88 | 0.212 | -0.88 | 0.209 | -0.87 | 0.22 | -0.55 | 0.423 | -0.89 | 0.21 | -0.89 | 0.229 | -0.74 | 0.287 |
|  | % the male | |  |  |  |  | 1.15 | 0.015 |  |  |  |  |  |  |  |  |  |  |  |  | -0.03 | 0.956 |
|  | % the poor | |  |  |  |  |  |  | 0.67 | 0.03 |  |  |  |  |  |  |  |  |  |  | 1.13 | 0.078 |
|  | % the elderly | |  |  |  |  |  |  |  |  | 0.78 | 0.001 |  |  |  |  |  |  |  |  | 0.86 | 0.009 |
|  | % the disabled | |  |  |  |  |  |  |  |  |  |  | 0.34 | 0.423 |  |  |  |  |  |  | -2.49 | 0.005 |
|  | N of divorces per 100,000 | |  |  |  |  |  |  |  |  |  |  |  |  | 0.03 | <0.001 |  |  |  |  | 0.03 | <0.001 |
|  | N of bars per 100,000 | |  |  |  |  |  |  |  |  |  |  |  |  |  |  | 0.01 | 0.719 |  |  | 0.1 | 0.1 |
|  | N of medical facilities per 100,000 | |  |  |  |  |  |  |  |  |  |  |  |  |  |  |  |  | 0.00 | 0.839 | 0.0 | 0.3 |
| Random  Part | Gu | N | 9 | | 9 | | 9 | | 9 | | 9 | | 9 | | 9 | | 9 | | 9 | | 9 | |
|  |  | Variance (SE) | 8.6(5.5) | | 8.8(5.6) | | 7.5(5.0) | | 6.2(4.5) | | 5.0(3.7) | | 7.6(5.3) | | 6.6(4.5) | | 8.5(5.5) | | 9.6(6.2) | | 6.2(4.4) | |
|  |  | VPC (%) | 6.5 | | 6.7 | | 5.8 | | 4.8 | | 4.0 | | 5.8 | | 5.5 | | 6.5 | | 7.3 | | 5.5 | |
|  |  | PCV (%) vs. M1 | - | | - | | 14.5 | | 29.5 | | 43.1 | | 13.7 | | 24.9 | | 3.7 | | -9.2 | | 29.0 | |
|  | Dong | N | 384 | | 384 | | 384 | | 384 | | 384 | | 384 | | 384 | | 384 | | 384 | | 384 | |
|  |  | Variance (SE) | 123.4(9.0) | | 122.8(9.0) | | 121.3(8.9) | | 122.1(8.9) | | 120.9(8.8) | | 122.9(9.0) | | 112.7(8.2) | | 122.9(9.0) | | 122.9(9.4) | | 107.1(8.2) | |
|  |  | VPC (%) | 93.5 | | 93.3 | | 94.2 | | 95.2 | | 96.0 | | 94.2 | | 94.5 | | 93.5 | | 92.7 | | 94.5 | |
|  |  | PCV (%) vs. M1 | - | | - | | 1.3 | | 0.6 | | 1.5 | | -0.1 | | 8.3 | | 0.0 | | 0.0 | | 12.8 | |
| Southwest Kown | | |  |  |  |  |  |  |  |  |  |  |  |  |  |  |  |  |  |  |  |  |
| Fixed  part | year | |  |  | 0.05 | 0.947 | 0.12 | 0.882 | 0.05 | 0.95 | 0.05 | 0.946 | 0.14 | 0.858 | 0.42 | 0.562 | 0.08 | 0.914 | 0.24 | 0.27 | 0.50 | 0.518 |
|  | % the male | |  |  |  |  | 0.96 | 0.008 |  |  |  |  |  |  |  |  |  |  |  |  | 0.63 | 0.069 |
|  | % the poor | |  |  |  |  |  |  | 1.41 | <0.001 |  |  |  |  |  |  |  |  |  |  | 0.65 | 0.354 |
|  | % the elderly | |  |  |  |  |  |  |  |  | 0.78 | 0.002 |  |  |  |  |  |  |  |  | 0.10 | 0.761 |
|  | % the disabled | |  |  |  |  |  |  |  |  |  |  | 1.98 | <0.001 |  |  |  |  |  |  | 0.97 | 0.366 |
|  | N of divorces per 100,000 | |  |  |  |  |  |  |  |  |  |  |  |  | 0.06 | <0.001 |  |  |  |  | 0.04 | <0.001 |
|  | N of bars per 100,000 | |  |  |  |  |  |  |  |  |  |  |  |  |  |  | 0.04 | <0.001 |  |  | 0.0 | <0.001 |
|  | N of medical facilities per 100,000 | |  |  |  |  |  |  |  |  |  |  |  |  |  |  |  |  | 0.00 | 0.22 | 0.0 | 0.9 |
| Random  part | Gu | N | 7 | | 7 | | 7 | | 7 | | 7 | | 7 | | 7 | | 7 | | 7 | | 7 | |
|  |  | Variance (SE) | 0(0) | | 0(0) | | 0(0) | | 0.2(1.4) | | 0(0) | | 0.5(1.5) | | 0.5(1.5) | | 0(0) | | 0(0) | | 0(0) | |
|  |  | VPC (%) | 0 | | 0 | | 0 | | 0.1 | | 0 | | 0.3 | | 0.4 | | 0 | | 0 | | 0 | |
|  |  | PCV (%)^*^ | **-** | | **-** | | N/A | | N/A | | N/A | | N/A | | N/A | | N/A | | N/A | | N/A | |
|  | Dong | N | 351 | | 351 | | 351 | | 351 | | 351 | | 351 | | 351 | | 351 | | 313 | | 313 | |
|  |  | Variance (SE) | 145.5(11.0) | | 145.5(11.0) | | 142.7(10.8) | | 134.8(10.3) | | 141.5(10.7) | | 135.1(10.3) | | 120.0(9.1) | | 138.5(10.5) | | 150.1(12.0) | | 111.1(8.9) | |
|  |  | VPC (%) | 100 | | 100 | | 100 | | 99.9 | | 100 | | 99.7 | | 99.6 | | 100 | | 100 | | 100 | |
|  |  | PCV (%)^*^ | **-** | | **-** | | 2.0 | | 7.4 | | 2.7 | | 7.2 | | 17.5 | | 4.8 | | -3.2 | | 23.6 | |
| Northwest Kown | | |  |  |  |  |  |  |  |  |  |  |  |  |  |  |  |  |  |  |  |  |
| Fixed  part | year | |  |  | 0.22 | 0.843 | 0.27 | 0.804 | 0.22 | 0.843 | 0.22 | 0.843 | 0.21 | 0.851 | 0.32 | 0.764 | 0.24 | 0.827 | 0.15 | 0.89 | 0.43 | 0.68 |
|  | % the male | |  |  |  |  | 0.29 | 0.707 |  |  |  |  |  |  |  |  |  |  |  |  | 1.83 | 0.068 |
|  | % the poor | |  |  |  |  |  |  | 0.14 | 0.869 |  |  |  |  |  |  |  |  |  |  | 0.21 | 0.867 |
|  | % the elderly | |  |  |  |  |  |  |  |  | 0.35 | 0.4 |  |  |  |  |  |  |  |  | 0.63 | 0.292 |
|  | % the disabled | |  |  |  |  |  |  |  |  |  |  | -0.16 | 0.862 |  |  |  |  |  |  | -2.73 | 0.078 |
|  | N of divorces per 100,000 | |  |  |  |  |  |  |  |  |  |  |  |  | 0.03 | 0.001 |  |  |  |  | 0.03 | 0.001 |
|  | N of bars per 100,000 | |  |  |  |  |  |  |  |  |  |  |  |  |  |  | 0.07 | 0.029 |  |  | 0.0 | 0.8 |
|  | N of medical facilities per 100,000 | |  |  |  |  |  |  |  |  |  |  |  |  |  |  |  |  | 0.02 | 0.012 | 0.0 | 0.1 |
| Random  part | Gu | N | 3 | | 3 | | 3 | | 3 | | 3 | | 3 | | 3 | | 3 | | 3 | | 3 | |
|  |  | Variance (SE) | 0(0) | | 0(0) | | 0(0) | | 0(0) | | 0(0) | | 0(0) | | 0(0) | | 0(0) | | 0(0) | | 0(0) | |
|  |  | VPC (%) | 0(0) | | 0(0) | | 0(0) | | 0 | | 0(0) | | 0 | | 0 | | 0(0) | | 0(0) | | 0(0) | |
|  |  | PCV (%) vs. M1 | **-** | | **-** | | N/A | | N/A | | N/A | | N/A | | N/A | | N/A | | N/A | | N/A | |
|  | Dong | N | 138 | | 138 | | 138 | | 138 | | 138 | | 138 | | 138 | | 138 | | 138 | | 138 | |
|  |  | Variance (SE) | 110.0(13.2) | | 109.9(13.2) | | 109.9(13.2) | | 109.9(13.2) | | 109.4(13.2) | | 109.9(13.2) | | 102.0(12.3) | | 106.3(12.8) | | 105.1(12.7) | | 93.9(11.3) | |
|  |  | VPC (%) | 100.0 | | 100.0 | | 100.0 | | 100.0 | | 100.0 | | 100.0 | | 100.0 | | 100.0 | | 100.0 | | 100.0 | |
|  |  | PCV (%) vs. M1 | **-** | | **-** | | 0.1 | | 0 | | 0.5 | | 0 | | 7.2 | | 3.3 | | 4.4 | | 14.6 | |

1) VPC: Variance partition coefficient, PCV: Proportional change in variance
