# Supplementary material for: The relative importance of macro versus micro geographical scale in explaining suicide variation in Seoul, South Korea 2014–2016
Source: PLoS One. 2022 Sep 9;17(9):e0273866. doi: 10.1371/journal.pone.0273866 (PMC9462743; doi:10.1371/journal.pone.0273866)
Supplement: S4 Table — (DOCX) [file pone.0273866.s004.docx]

Table S4. Bivariate and fully- adjusted regression when adjusted for the number of hospital-level medical institutions

|  | **Variable** | | Year stratified | | | | | | Pooled | | Kown stratified | | | | | | | | | | |
| --- | --- | --- | --- | --- | --- | --- | --- | --- | --- | --- | --- | --- | --- | --- | --- | --- | --- | --- | --- | --- | --- |
|  |  |  | 2014 | | 2015 | | 2016 | | Year pooled | | Downtown | | East-south | | East-north | | West-south | | West-north | | |
|  |  |  | b | P value | b | P value | b | P value | b | P value | b | P value | b | P value | b | P value | b | P value | b | P value |  |
| Fixed  part | year | |  |  |  |  |  |  | -0.27 | 0.544 | -2.43 | 0.14 | -0.37 | 0.711 | -0.67 | 0.314 | 0.51 | 0.486 | 0.41 | 0.692 |  |
|  | % the male | | 0.30 | 0.437 | -0.40 | 0.331 | 0.52 | 0.16 | 0.06 | 0.786 | 1.11 | 0.242 | 2.88 | <0.001 | 0.19 | 0.686 | 0.56 | 0.102 | 1.32 | 0.175 |  |
|  | % the poor | | 1.99 | <0.001 | 2.94 | <0.001 | 2.13 | <0.001 | 2.47 | <0.001 | 0.99 | 0.504 | 5.43 | <0.001 | 1.47 | 0.017 | 0.53 | 0.433 | -0.32 | 0.791 |  |
|  | % the elderly | | 0.08 | 0.798 | 0.16 | 0.601 | 0.23 | 0.411 | 0.21 | 0.243 | -1.63 | 0.026 | 0.51 | 0.257 | 0.80 | 0.013 | 0.00 | 0.99 | 0.58 | 0.342 |  |
|  | % the disabled | | -3.14 | <0.001 | -3.84 | <0.001 | -3.10 | <0.001 | -3.60 | <0.001 | -5.71 | 0.004 | -7.11 | <0.001 | -2.78 | 0.001 | 1.12 | 0.277 | -1.96 | 0.191 |  |
|  | N of divorces per 100,000 | | 0.04 | <0.001 | 0.06 | <0.001 | 0.03 | <0.001 | 0.05 | <0.001 | 0.01 | 0.521 | 0.07 | <0.0010 | 0.03 | <0.001 | 0.04 | <0.001 | 0.03 | 0.002 |  |
|  | N of bars per 100,000 | | 0.01 | 0.006 | 0.02 | <0.001 | 0.01 | 0.031 | 0.01 | <0.001 | 0.02 | <0.001 | 0.00 | 0.9 | 0.01 | 0.6 | 0.03 | <0.001 | 0.08 | <0.001 |  |
|  | N of hospital per 100,000 | | 0.12 | 0.312 | 0.05 | 0.702 | 0.76 | <0.001 | 0.39 | <0.001 | 0.19 | 0.262 | 0.54 | 0.0 | 0.19 | 0.2 | 0.07 | 0.6 | 0.25 | 0.5 |  |
| Random  Part* | Level 2  Gu | N | 24 | | 25 | | 25 | | 25 | | 3 | | 4 | | 8 | | 7 | | 3 | | |
|  |  | Variance estimate (SE) | 6.8(4.4) | | 0(0) | | 0(0) | | 2.3(1.7) | | 7.9(12.2) | | 0(0) | | 5.0(3.8) | | 0(0) | | 0(0) | | |
|  |  | % variance attributable | 5.0 | | 0 | | 0 | | 1.4 | | 3.1 | | 0 | | 4.4 | | 0 | | 0 | | |
|  |  | % explained vs. M1 | 37.7 | | N/A | | N/A | | 72.5 | | 79.2 | | 100 | | 43.1 | | N/A | | N/A | | |
|  | Level 1  Dong | N | 402 | | 421 | | 424 | | 1247 | | 141 | | 253 | | 384 | | 331 | | 138 | | |
|  |  | Variance estimate (SE) | 127.8(9.93 | | 190.9(13.2) | | 168.4(10.9) | | 165.5(6.7) | | 249.6(30.1) | | 169.8(15.1) | | 108.4(7.9) | | 110.1(8.6) | | 95.7(11.5) | | |
|  |  | % variance attributable | 95.0 | | 100 | | 100 | | 98.6 | | 96.9 | | 100 | | 95.6 | | 100.0 | | 100.0 | | |
|  |  | % explained vs. M1 | 34.9 | | 36.4 | | 35.7 | | 31.7 | | 36.5 | | 68.5 | | 11.7 | | 24.4 | | 13.0 | | |

*M1: Model adjusted for year.
